# Novel cerebrovascular pathology in mice fed a high cholesterol diet

**DOI:** 10.1186/1750-1326-4-42

**Published:** 2009-10-24

**Authors:** Sonia Franciosi, Miguel A Gama Sosa, Daniel F English, Elizabeth Oler, Twethida Oung, William GM Janssen, Rita De Gasperi, James Schmeidler, Dara L Dickstein, Christoph Schmitz, Sam Gandy, Patrick R Hof, Joseph D Buxbaum, Gregory A Elder

**Affiliations:** 1 Department of Psychiatry, Mount Sinai School of Medicine, One Gustave L Levy Place, New York, NY 10029, USA; 2 Laboratory of Molecular Neuropsychiatry, Mount Sinai School of Medicine, One Gustave L Levy Place, New York, NY 10029, USA; 3 Research and Development Service, James J Peters Department of Veterans Affairs Medical Center, 130 West Kingsbridge Road, Bronx, NY 10468, USA; 4 Department of Neuroscience, Mount Sinai School of Medicine, One Gustave L Levy Place, New York, NY 10029, USA; 5 Computational Neurobiology and Imaging Center, Mount Sinai School of Medicine, New York, NY 10029, USA; 6 Department of Neurology, Mount Sinai School of Medicine, One Gustave L Levy Place, New York, NY 10029, USA; 7 Neurology Service, James J Peters Department of Veterans Affairs Medical Center, 130 West Kingsbridge Road, Bronx, NY 10468, USA; 8 Department of Geriatrics and Adult Development, Mount Sinai School of Medicine, One Gustave L Levy Place, New York, NY 10029, USA; 9 Department of Genetics and Genomic Sciences, Mount Sinai School of Medicine, New York, NY 10029, USA

## Abstract

**Background:**

Hypercholesterolemia causes atherosclerosis in medium to large sized arteries. Cholesterol is less known for affecting the microvasculature and has not been previously reported to induce microvascular pathology in the central nervous system (CNS).

**Results:**

Mice with a null mutation in the low-density lipoprotein receptor (LDLR) gene as well as C57BL/6J mice fed a high cholesterol diet developed a distinct microvascular pathology in the CNS that differs from cholesterol-induced atherosclerotic disease. Microvessel diameter was increased but microvascular density and length were not consistently affected. Degenerative changes and thickened vascular basement membranes were present ultrastructurally. The observed pathology shares features with the microvascular pathology of Alzheimer's disease (AD), including the presence of string-like vessels. Brain apolipoprotein E levels which have been previously found to be elevated in LDLR-/- mice were also increased in C57BL/6J mice fed a high cholesterol diet.

**Conclusion:**

In addition to its effects as an inducer of atherosclerosis in medium to large sized arteries, hypercholesterolemia also induces a microvascular pathology in the CNS that shares features of the vascular pathology found in AD. These observations suggest that high cholesterol may induce microvascular disease in a range of CNS disorders including AD.

## Background

Cholesterol is essential for building and maintaining cell membranes. Many steroid hormones also require cholesterol as a precursor. Yet, despite its role in essential biochemical processes and support of membrane structure, hypercholesterolemia is associated with negative health outcomes especially its association with vascular disease. In particular the level of serum low-density lipoprotein cholesterol (LDL-C) is a key risk factor for atherosclerosis and lowering LDL-C significantly reduces the risk of coronary heart disease [1,2].

Serum cholesterol has also been suggested as a risk factor for or modulator of neurological diseases although the effects appear complex and disease specific. In Alzheimer's disease (AD) much attention has focused on how cholesterol influences the enzymes which process the amyloid precursor protein (APP) and in particular that high cellular cholesterol shifts APP processing towards production of the amyloid β peptide (Aβ), which in turn accumulates in neuritic plaques, while lower cellular cholesterol levels promote α-secretase cleavage of APP and prevent Aβ formation [3]. In contrast, higher serum cholesterol levels have been suggested to be associated with a lower risk of Parkinson's disease [4], while low serum LDL-C levels have been associated with worsening of amyotrophic lateral sclerosis [5]. Curiously, despite hypercholesterolemia's well-established role in promoting ischemic heart disease, serum cholesterol is not a strong risk factor for ischemic stroke [2,6] or vascular dementia [7].

How cholesterol modulates susceptibility to neurological diseases is incompletely understood. Hypercholesterolemia is best known for producing atherosclerosis in relatively large arteries such as the aorta or coronary arteries [8]. In contrast, hypercholesterolemia is not normally thought of as affecting microvessels pathologically even though a substantial literature exists showing that high cholesterol adversely affects the physiological functioning of the microvasculature including microvessels in the brain [9].

Here we report that mice fed a high cholesterol diet develop a vascular pathology that affects the CNS microvasculature. This pathology is distinctive from cholesterol-induced atherosclerotic disease and shares some features of the microvascular pathology associated with AD [10]. These findings have implications for the role that cholesterol may play in inducing vascular disease in a variety of neurological diseases including AD.

## Results

### Experimental design for manipulation of plasma cholesterol in LDLR-/- mice

C57BL/6J mice fed a high cholesterol diet develop at most mild elevations in plasma cholesterol. LDLR-/- mice fed a standard low cholesterol rodent chow diet (~4-6% fat, 0.04% or less cholesterol) also have only moderately increased plasma cholesterol levels with a slightly increased susceptibility to atherosclerosis [11-13]. However, by increasing dietary cholesterol to 0.15% or greater [14], plasma cholesterol in LDLR-/- mice increases dramatically and within 16 weeks of dietary modification the mice develop extensive atherosclerotic lesions in the aortic root [15,16].

We have previously utilized the ability to modulate plasma cholesterol levels in LDLR-/- mice to test the effects of relatively short term modulation of plasma cholesterol (16 weeks on diet) on brain Aβ production and behavior in LDLR-/- mice [17,18]. Hypercholesterolemia has a well-established role in promoting atherosclerosis, for example in the coronary arteries. Less is known about how cholesterol affects the brain vasculature and indeed serum cholesterol is not regarded as a major risk factor for ischemic stroke [2,6] or vascular dementia [7]. We therefore determined whether short term (16 weeks) or long term (10 months) modulation of dietary cholesterol induces brain vascular pathology. Beginning at two months of age, we fed C57BL/6J wild type mice and LDLR-/- mice either low (LCD, standard laboratory chow) or high cholesterol (HCD, 0.15% cholesterol) diets for 16 weeks or 10 months and examined the brains for vascular pathology at the end of treatment.

**Figure 1:**
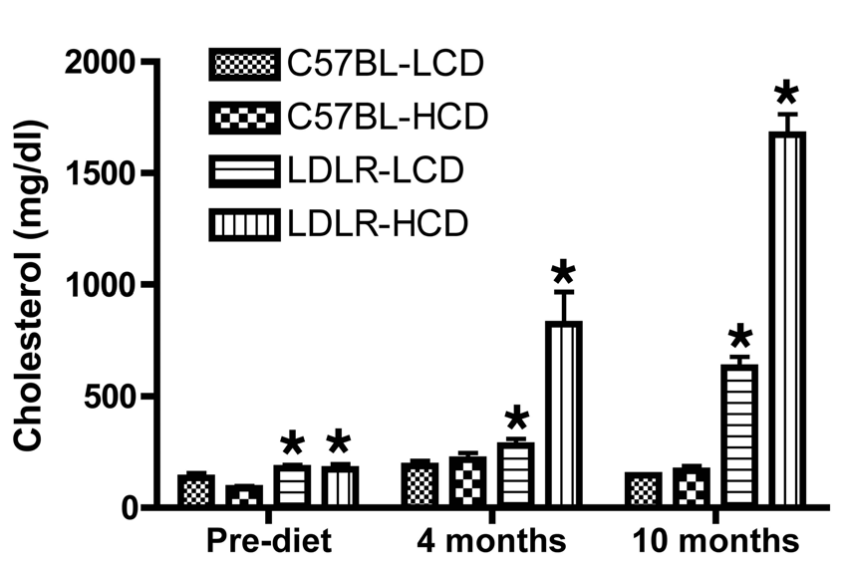
**Plasma cholesterol levels in C57BL6/J and LDLR-/- mice fed low- or high-cholesterol diets**. Two-month old C57BL/6J wild type (C57BL) or LDLR-/-mice were fed either low (LCD) or high (HCD) cholesterol diets for 4 or 10 months. Plasma cholesterol was determined at baseline (Pre-diet, n = 11/group), as well as after 4 (n = 11/group; except n = 12, LDLR-HCD) or 10 (n = 6, C57BL-LCD; n = 6, C57BL-HCD; n = 5, LDLR-LCD; and n = 2, LDLR-HCD) months. Asterisks indicate groups different from C57BL-LCD at each time point (p < 0.05). Results are discussed further in the text.

To verify the effects of dietary treatment, we measured total plasma cholesterol pre- and post-treatment (Figure 1). As in our previous studies [17,18], we found that baseline plasma cholesterol at two months of age was modestly elevated in LDLR-/- mice (~160 mg/dl) compared to C57BL/6J mice (~100 mg/dl, p = 0.0008, unpaired Student's *t *test). Following 16 weeks of dietary treatment, plasma cholesterol was ~825 mg/dl in the LDLR-/- mice fed the HCD compared to ~280 in LDLR-/- mice continued on the LCD. Plasma cholesterol in C57BL/6J mice ranged from 188 mg/dl in mice fed the LCD diet to 216 mg/dl in mice fed the HCD. A two-way ANOVA revealed the expected effects of both diet (F_1,41 _= 12.89, p = 0.0009) and genotype (F_1,41 _= 19.28, p < 0.0001) and a significant interaction of diet and genotype (F_1,41 _= 10.54, p = 0.0023) with plasma cholesterol increased in LDLR-HCD mice compared to all of the other groups (p < 0.001, Tukey HSD). Despite 16 weeks on the HCD, plasma cholesterol in C57BL/6J mice was unchanged compared to C57BL/6J mice on the LCD.

Comparing pre- to post-16-week treatment values, a repeated measures two-way ANOVA revealed effects of group (F_3,41 _= 17.43, p < 0.0001) and time (F_1,41 _= 38.31, p < 0.0001), as well as a significant interaction effect (F_3,41 _= 11.33, p < 0.0001) with comparisons between groups showing that only in the LDLR-HCD group was plasma cholesterol significantly changed from baseline (p < 0.001, Tukey HSD).

In the cohort of animals that was treated for 10 months, plasma cholesterol was higher than 1,600 mg/dl in the LDLR-/- mice fed the HCD compared to ~630 mg/dl in LDLR-/- mice continued on the LCD. Two-way ANOVA revealed effects of both diet (F_1,16 _= 226.7, p < 0.0001) and genotype (F_1,16 _= 795.2, p < 0.0001) and a significant interaction of diet and genotype (F_1,16 _= 210.1, p < 0.0001) with plasma cholesterol increased in LDLR-HCD compared to other groups (p = 0.04 vs. C57BL-LCD and C57BL-HCD; p = 0.06 vs. LDLR-L, unpaired *t *test with Welch correction). LDLR-LCD was also increased vs. both C57BL/6J groups (p < 0.0007). Interestingly, 10 months on a HCD resulted in no change in cholesterol levels in C57BL/6J mice with plasma cholesterol averaging 145 mg/dl in mice fed the LCD and 165 mg/dl in mice fed the HCD (p = 0.4117).

### Microvascular pathology in mice fed a high cholesterol diet

**Figure 2:**
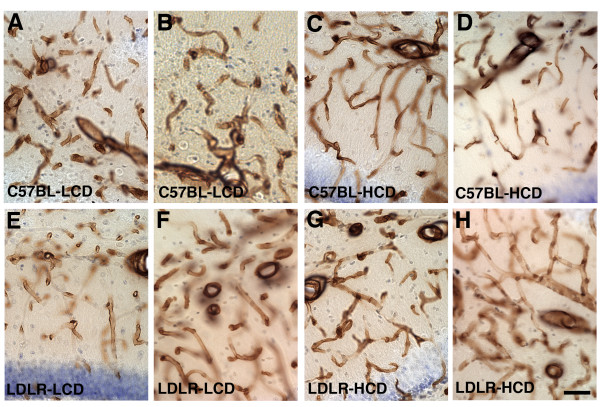
**Vascular pathology in C57BL/6J and LDLR-/- mice fed a high-cholesterol diet**. Anti-collagen IV immunoperoxidase-stained microvessels in the hippocampus of one-year old C57BL/6J (A-D), or LDLR-/- mice (E-H) fed low-cholesterol (A, B, E, F), or high-cholesterol (C, D, G, H) diets for 10 months. Mice in the high-cholesterol fed groups (C57BL-HCD and LDLR-HCD) exhibit a mixture of microvessels that are often thinner and irregular (see especially panels C, D) while other microvessels appear enlarged (see especially panels F, H). Scale bar = 50 μm.

**Figure 3:**
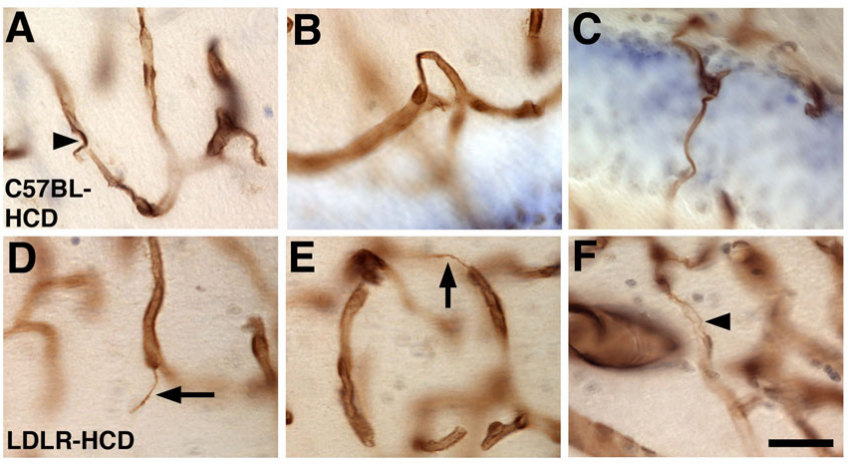
**Abnormal vascular morphologies in C57BL/6J and LDLR-/- mice fed a high- cholesterol diet**. Anti-collagen IV immunoperoxidase-stained microvessels in the hippocampus of one year old C57BL/6J (A, B, C), or LDLR-/- mice (D, E, F) fed a HCD for 10 months. Degenerating vascular segments are indicated by arrowheads in panels A and F. Typical string vessels are indicated by arrows in panels D and E. Panels B and C show dysmorphic and abnormally twisted vessels. Scale bar = 50 μm.

We examined the brains of six month old or one-year old mice fed HCD or LCD diets for 4 or 10 months respectively using collagen IV immunostaining to visualize the vasculature. At both ages, microvessels in C57BL-HCD, LDLR-LCD, and LDLR-HCD exhibited pathological changes. Examples of pathological vessels in the hippocampus are shown in Figure 2 for mice fed these diets for 10 months. Microvessels in the C57BL-LCD mice exhibited generally smooth contours and were regular in appearance. In contrast, mice in the high cholesterol-fed groups (C57BL-HCD and LDLR-HCD) exhibited a mixture of microvessels that were often thinner and irregular while other microvessels appeared enlarged. In addition microvessels in C57BL-HCD and LDLR-HCD mice exhibited a variety of abnormal morphologies including string-like vessels while other vessels displayed a kinked or twisted morphology (Figure 3). These changes were found in both cortical and subcortical regions and, whereas present in 6-month old mice fed the experimental diets for four months, they were more apparent in one-year old mice that had received the diets for 10 months. The thinning and irregularity as well as the strings and twisted morphologies are suggestive of a degenerative process and are highly reminiscent of the vascular pathology seen in AD [10,19].

**Figure 4:**
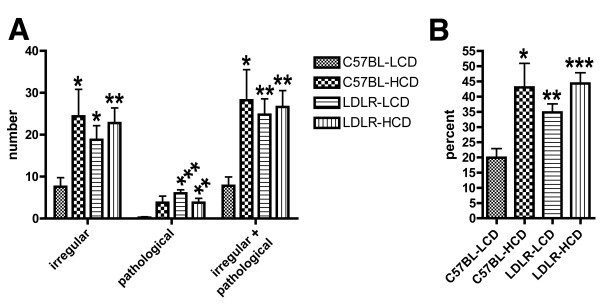
**Quantification of pathological microvessels in mice fed low- or high-cholesterol diets**. The number of irregular and pathological microvessels was assessed in the hippocampus of C57BL/6J and LDLR-/- mice fed low- or high-cholesterol diets for 10 months (n = 5 per group). Microvessels were classified as normal, irregular, abnormally dilated, tortuous, or string-like. An average of 57 ± 4.6 microvessels were scored per animal. Abnormally dilated, tortuous and string vessels were summed as ''pathologic''. In panel (A), the average number of irregular, pathological or irregular + pathological microvessels per hippocampus is presented. In panel (B), the number of irregular + pathological vessels divided by the total number of vessels counted is presented. Comparisons were made between each experimental group and C57BL-LCD mice using unpaired *t *tests with the test of significance according to the Holm procedure. Asterisks indicate: * p = 0.01-0.05; ** p = 0.001-0.01; *** p < 0.001.

To quantify the number of pathological microvessels in the hippocampus of C57BL/6J and LDLR-/- mice fed low or high cholesterol diets for 10 months, microvessels were selected using a systematic-random stereologic sampling methodology and visually classified as normal, irregular, or abnormally dilated, tortuous, or string-like. As shown in Figure 4, compared to C57BL-LCD mice, there were more irregular as well as pathologic vessels in C57BL-HCD, LDLR-LCD, and LDLR-HCD mice.

### Increased microvessel diameter in mice fed a high cholesterol diet

**Figure 5:**
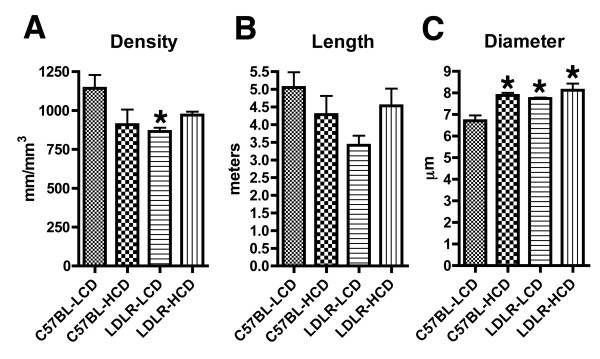
**Quantification of microvascular parameters in mice fed low- or high-cholesterol diets**. Vascular parameters were assessed stereologically in the hippocampus of C57BL/6J and LDLR-/- mice fed low- or high-cholesterol diets for 10 months. Quantitative analyses were performed on collagen IV immunoperoxidase-stained sections (n = 6 per group). Data are presented as total vascular density (A), total vessel length per hippocampus (B), and average microvessel diameter (C). Asterisks indicate values that are statistically different from C57BL-LCD (p < 0.05, Tukey HSD).

To determine whether quantitative vascular parameters were altered, we performed stereologic assessments measuring vascular length, length density, and diameter on collagen IV-immunostained vessels in the hippocampus of animals fed the diets for 10 months. The most consistent change quantitatively was that microvessel diameter was increased in C57BL-HCD, as well as LDLR-/- fed either the LCD or HCD. As shown in Figure 5, mean diameters increased from 6.7 ± 0.24 μm in C57BL-LCD to 7.8 ± 0.12 μm in C57BL-HCD, 7.7 ± 0.05 μm in LDLR-LCD and 8.1 ± 0.31 μm in LDLR-HCD (F_1,20 _= 14.00, p = 0.0013 for diet; F_1,20 _= 9.319, p = 0.0063 for genotype; F_1,20 _= 3.554, p = 0.074 for interaction; p < 0.015, C57BL-HCD, LDLR-LCD and LDLR-HCD vs. C57BL-LCD, Tukey HSD; no significant differences between C57BL-HCD, LDLR-LCD and LDLR-HCD groups). Compared to controls where microvessel length density was 1,142 ± 86 mm/mm^3 ^(± SEM), the length density was reduced by 21% in C57BL-HCD (909 ± 97), by 23% in LDLR-LCD (879 ± 26) and by 15% in LDLR-HCD (971 ± 22) with a two-way ANOVA revealing an interaction effect of diet and genotype (F_1,20 _= 0.9222, p = 0.34 for diet; F_1,20 _= 2.612, p = 0.12 for genotype; F_1,20 _= 6.435, p = 0.02 for interaction), although the only significant group difference was that LDLR-LCD was reduced compared to C57BL-LCD (p = 0.038, Tukey HSD). There were no differences between the groups in total microvessel length (p > 0.05 all group comparisons, Tukey HSD) although a two-way ANOVA revealed an interaction effect of diet and genotype (F_1,20 _= 0.1576, p = 0.6956 for diet; F_1,20 _= 2.461, p = 0.1324 for genotype; F_1,20 _= 4.562, p = 0.0452 for interaction). Thus the most striking change quantitatively was that hypercholesterolemic LDLR-/- mice as well as C57BL/6J mice fed a HCD exhibit increased microvessel diameters.

### Ultrastructural analysis of microvascular pathology

**Figure 6:**
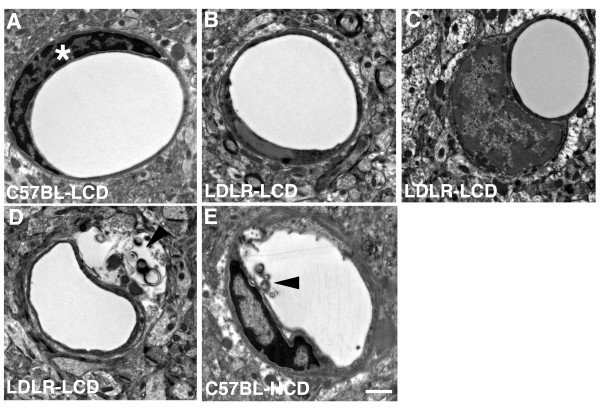
**Ultrastructural analysis of the cerebral microvasculature in mice fed low- or high- cholesterol diets**. Transversely sectioned capillaries are shown from the entorhinal cortex of C57BL/6J or LDLR-/- mice fed low- (LCD) or high- (HCD) cholesterol diets for 4 months. The microvessel in panel A from a C57BL/6J mouse fed a LCD exhibits a circular lumen, an intact endothelial cell, and smooth capillary walls. Note the altered chromatin structure in the endothelial cell nucleus in panel B, and the swollen endothelial cell nucleus in panel C. A normal endothelial cell nucleus is indicated by an asterisk in A. Arrowheads indicate an expanded extravascular space containing amorphous debris (D), or luminal degeneration (E) in an LDLR-/- mouse fed a LCD (D), or a C57BL/6J mouse fed a HCD (E). Scale bar = 1 μm.

**Figure 7:**
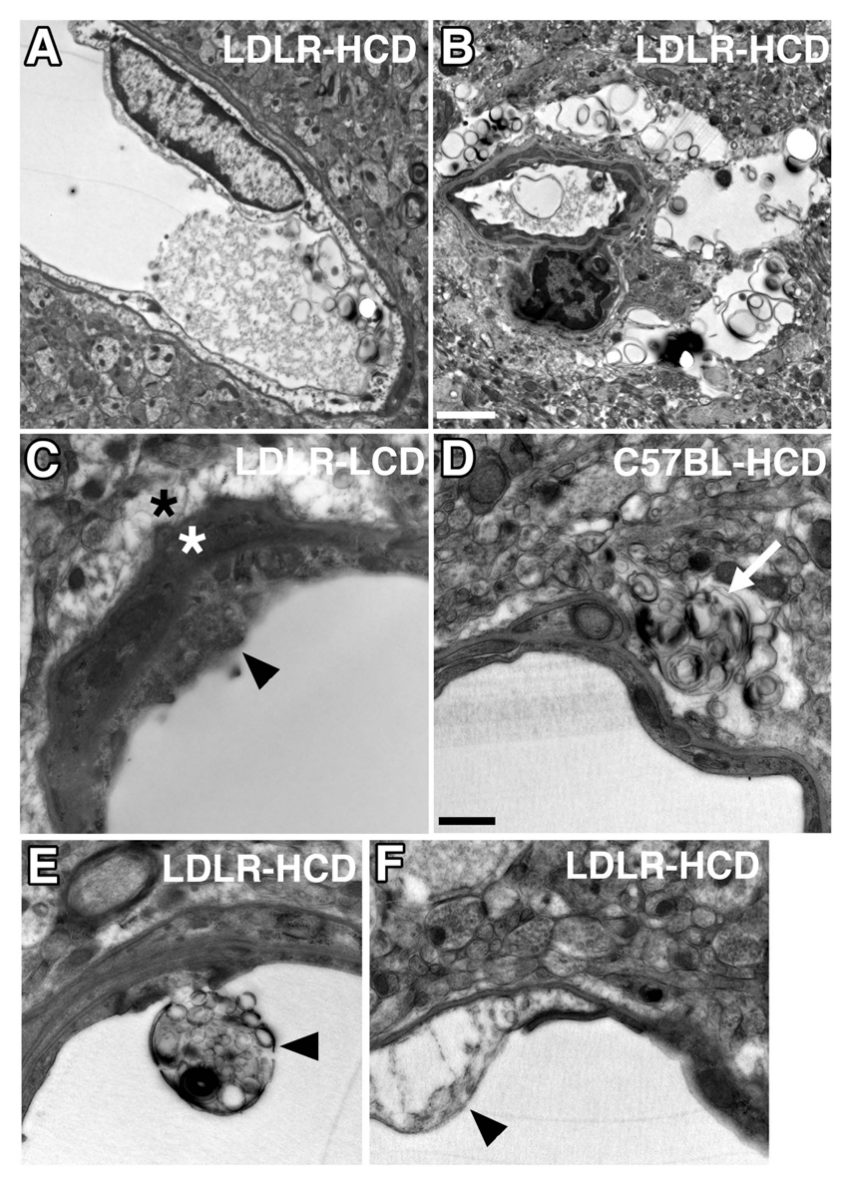
**Ultrastructural analysis of microvascular pathology in LDLR-/- and C57BL/6J mice**. Additional examples of microvascular pathology from LDLR-HCD (A, B, E, F), LDLR-LCD (C), and C57BL-HCD (D) are shown. In A and B, note the severely degenerating microvessels in an LDLR mouse fed a high cholesterol diet. In C, note the thickened basal laminae (white asterisk), expanded perivascular space (black asterisk), and degenerating capillary wall (arrowhead) in an LDLR-/- mouse fed a LCD. In D, an arrow indicates an expanded perivascular space filled with amorphous debris in a C57BL/6J mouse fed a HCD. Examples of degeneration in the capillary wall in LDLR-HCD mice are indicated by arrowheads in panels E and F. Scale bar in B = 2 μm for panels A and B; scale bar in D = 500 nm for panels C-F.

To determine the ultrastructural basis of the pathology we examined the microvasculature by electron microscopy. We examined animals after 16 weeks of dietary treatment, as at that time, at the light microscopy level, microvascular pathology was already evident. Figures 6 and 7 show examples of microvessels in the entorhinal cortex from each group. Microvessels in the C57BL-LCD mice exhibited classic neurovascular ultrastructure with circular lumens, intact endothelial cells and smooth capillary walls (Figure 6A). In contrast, microvessels in C57BL-HCD, LDLR-LCD, and LDLR-HCD groups showed varying degrees of pathology. The endothelial cell nuclear chromatin often appeared amorphous (Figure 6B) with in some cases the endothelial cell nuclei distorted and sometimes swollen (Figure 6C). In addition, luminal circularity was frequently lost (Figure 6D) and there were varying degrees of capillary wall degeneration (Figure 6E).

Figures 7A and 7B show examples of advanced degenerative changes in microvessels from an LDLR-HCD mouse. Accompanying these changes, the vascular basal laminae (Figure 7C) were often thickened and expanded perivascular spaces containing amorphous debris were sometimes apparent (Figure 7C, D). Similar changes were also apparent in C57BL-HCD mice (Figures 6E, 7D).

### ApoE protein levels in brain are increased in C57BL/6J mice fed a high cholesterol diet

**Figure 8:**
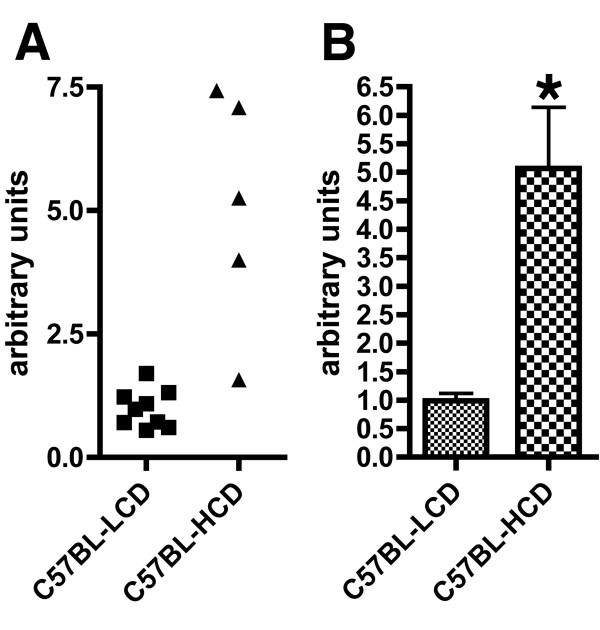
**Expression of ApoE in brain of C57BL/6J mice fed a high-cholesterol diet**. Shown are levels of ApoE determined by quantitative Western blotting on brain homogenates from C57BL/6J fed a HCD (n = 5) diet for 16 weeks beginning at 2 months of age compared to C57BL/6J maintained on a LCD (n = 9). Data are shown plotted as individual values (A) or as summed values ± SEM (B). Asterisk indicates p = 0.0002 vs. control (unpaired Student's *t *test).

Plasma ApoE is elevated in LDLR-/- mice and ApoE levels rise in the presence of hypercholesterolemia [12]. We [17] and others [20] have reported that ApoE levels are elevated in the brain of LDLR-/- mice. To determine whether ApoE levels might be modulated in brain in C57BL/6J mice fed a HCD as well, we determined by Western blotting levels of ApoE in the cortex of C57BL/6J mice fed a HCD for 16 weeks. As shown in Figure 8, compared to laboratory chow-fed mice, ApoE levels were increased approximately 5-fold in mice fed the HCD (p = 0.0002, unpaired Student's *t *test). Thus brain ApoE levels are dramatically increased in C57BL/6J mice fed a HCD diet.

## Discussion

Here we describe a novel cerebrovascular pathology associated with feeding a high cholesterol diet to C57BL/6J wild type or LDLR-/- mice. The pathology includes a variety of abnormal vascular morphologies including twisted vessels and string vessels. Stereologic assessments confirmed a visual impression that microvessels are larger in high cholesterol fed mice and revealed a tendency towards a decreased vascular density in mice fed high cholesterol diets for 10 months. At the ultrastructural level, microvessels showed varying degrees of endothelial cell pathology including altered nuclear chromatin structure and nuclear swelling as well as degenerative changes in the luminal wall. There was also thickening of the vascular basal laminae and expanded perivascular spaces often filled with amorphous debris. All of these features are indicative of a degenerative process and have not been previously described as being part of the spectrum of cholesterol related vascular pathology.

LDLR-/- mice spontaneously develop moderately increased total plasma cholesterol levels and increased LDL-C when fed a standard low cholesterol rodent chow diet (~4-6% fat, 0.04% or less cholesterol). When fed a high cholesterol diet, LDLR-/- mice develop massively increased both total plasma cholesterol as well as LDL-C [14]. On a standard lab chow diet, nearly all plasma cholesterol in C57BL/6J mice is HDL-C [21]. However, when fed a high cholesterol diet such as that used here, most of the plasma cholesterol in C57BL/6J mice becomes LDL-C and the ratio of LDL-C to HDL-C becomes inverted compared to mice on a lab chow diet [22]. Thus, although we do not have LDL-C and LDL-H levels available on the mice in the present study, we suspect that the pathology observed in C57BL/6J fed a high cholesterol diet likely reflects increased LDL-C despite normal total plasma cholesterol levels.

Hypercholesterolemia is best known for its role in atherosclerotic vascular disease, a process that is most apparent in larger arteries such as the aorta or the coronary arteries. Cholesterol is generally not thought to cause pathology in the microvasculature. However, despite the lack of reported morphological changes a substantial literature has shown that high cholesterol/high fat diets induce microvascular dysfunction [23-29]. In arterioles, this dysfunction takes the form of impaired responses to stimuli that induce vasodilation, while in postcapillary venules it is manifested as increased leukocyte and platelet adhesion. For example in hypercholesterolemic LDLR-/- mice following ischemia/reperfusion injury, larger numbers of adherent leukocytes are seen in postcapillary venules and vascular permeability is increased [27]. A low-grade inflammatory response occurs in association with these functional changes [9]. In experimental animals, functional changes can be seen in the microvasculature before arterial lesions develop and occur in multiple tissues including the brain [9,25,26]. This report is to our knowledge the first to describe pathologic changes in the microvasculature associated with a high cholesterol diet.

Along with cholesterol, hypertension, and diabetes are also potent cardiovascular risk factors for atherosclerosis in medium to large sized arteries and both are associated with microvascular pathology. In hypertension, the microvascular pathology takes the form of a hyaline thickening of arteriolar walls referred to hyaline arteriosclerosis or a hyperplastic change in which a laminated thickening of the vessel walls occurs due to smooth muscle cell proliferation as well as thickening and reduplication of the basement membrane, a process referred to as hyperplastic arteriosclerosis [8]. Both conditions can produce progressive luminal narrowing. However, the changes described here do not resemble either form of hypertension related vascular pathology.

Diabetes may also be associated with hyaline arteriolosclerosis [30]. However, diabetes produces its own distinctive microangiopathy, one of the most consistent features of which involves diffuse thickening of the vascular basement membrane [30]. This form of microangiopathy is seen in capillaries in a variety of tissues and is regarded as underlying the development of diabetic nephropathy, retinopathy, and some types of peripheral neuropathy. The pathology described here shares some features with diabetic microangiopathy, in terms of thickened vascular membranes and indeed C57BL/6 mice fed a high-fat Western diet develop hyperglycemia and insulin resistance [31-33], raising the possibility that diet induced insulin resistance might be contributing to the vascular pathology observed here. Arguing against this possibility, however, is the fact that C57BL/6 mice develop insulin resistance consistently only on diets containing more than 20% fat [31-33] and do not develop insulin resistance on lower-fat atherogenic diets [33]. C57BL/6 mice fed a 1% cholesterol-enriched diet without increased fat (4.4%), similar to that used here also do not become insulin resistant [34]. LDLR-/- mice fed a high-fructose diet in addition do not become insulin resistant, despite the diet elevating plasma cholesterol levels to those as high as seen with high-fat diets [32]. Thus, wild type C57BL/6 mice fed the type of high-cholesterol diet used in the present study (0.15% cholesterol/4.3% fat) do not typically develop insulin resistance, making it unlikely that the vascular pathology observed here can be ascribed to insulin resistance.

Interestingly, some of the pathological alterations described bear a resemblance to the vascular pathology associated with AD. Whereas it seems that no simple correlation exists between serum cholesterol levels and the risk of developing AD [7,35,36], elevated midlife cholesterol has been associated with an increased risk of AD [7]. In addition, higher total serum cholesterol and LDL-C correlate with a more rapid cognitive decline in patients with AD [37]. Dyslipidemia is also a component of the metabolic syndrome along with obesity, hypertension, and hyperglycemia. There has been recently much interest in the role that the individual components of the metabolic syndrome may play in the development of AD as well as other dementias [38-40], with some studies suggesting that age-related cognitive impairment is more likely to develop when the metabolic syndrome is present [41].

AD is accompanied by vascular pathology. In the most recognized form of this pathology, cerebral amyloid angiopathy, amyloid is deposited the walls of blood vessels with leptomeningeal and neocortical arteries and arterioles being most affected [42]. Vascular pathology, however, also occurs in the microvasculature leading to a decreased density and fragmentation of microvasculature [10,19]. Microvessels appear less branched and thin atrophic vessels known as string vessels appear while other vessels become kinked and looped. The cause and relationship of vascular pathology to cognitive decline in AD remains unclear although patients with Down syndrome display a similar vascular pathology that is present in young cases devoid of neuritic plaques and neurofibrillary tangles [19], arguing that vascular changes may precede the development of these lesions. Alterations of the vascular basement membranes, in particular thickening of basement membranes, have been suggested to be an early feature of the microvascular pathology in AD [43]. Similarities between the cholesterol related pathology described here and the microvascular pathology of AD include the presence of string vessels, tortuous and looped vessels, and thickened basement membranes. The pathology observed here however differs from AD by the presence of increased microvessel diameters.

How a high-cholesterol diet induces microvascular pathology in brain is not known. The generation of reactive oxygen species, in particular superoxide is thought to be a major factor in cholesterol's effects on dysfunction of the microvasculature [9]. Upregulation of cell adhesion molecules including intercellular adhesion molecule-1 (ICAM-1) and P-selectin on the endothelium along with the release of additional cytokines from circulating lymphocytes likely underlie the increased leukocyte and platelet adhesion, and create a generally proinflammatory state [9]. This effect is seen in experimental animals in ranges of plasma cholesterol that are only modestly elevated. Increased proinflammatory cytokines, microglial reaction, and astrogliosis have also been seen in the brains of LDLR-/- mice following high-cholesterol/high-fat diets [44]. However, based on immunohistochemical staining we have not seen any obvious microglial or astroglial reaction in LDLR-/- mice fed a selective high-cholesterol diet (unpublished observations). Clearly further studies will be needed to delineate the molecular mechanisms underlying the microvascular pathology induced by a high-cholesterol diet.

ApoE plays a significant role in modulating cholesterol transport in the periphery as well as the brain where it is known to affect for example amyloid deposition [45]. Previously we [17] and others [20] have reported that ApoE levels are elevated in the brain of LDLR-/- mice. Here we show that ApoE levels in brain are also increased in C57BL/6J mice fed a HCD. Altered ApoE levels in brain may in addition modify responses to disease processes in the CNS.

## Conclusion

Future studies will be necessary to elucidate the exact mechanisms that underlie the cerebral microvascular pathology associated with elevated cholesterol. However, collectively, these studies show that mice fed a high-cholesterol diet develop a distinctive CNS microvasculature pathology. These findings have implications for the role that cholesterol related vascular disease might play in neurological diseases including AD.

## Materials and methods

### Mice

Male LDLR-/- mice were purchased from Jackson Laboratories (Bar Harbor, MA; Ldlr KO stock # 002207 strain name B6.12957-Ldlr^tm1Her^). These mice were originally generated using a 129 ES cell line and have been backcrossed for 10 generations onto the C57BL/6J background. Age-matched male C57BL/6J wild type mice also obtained from Jackson Laboratories were used as controls. Animals were housed on 12 h light/dark cycles. All protocols were approved by the Mount Sinai School of Medicine Institutional Animal Care and Use Committee and were in conformance with the National Institutes of Health "Guide for the Care and Use of Laboratory Animals".

### Dietary manipulations

LDLR-/- and C57BL/6J control mice were maintained on a standard low-cholesterol rodent chow diet containing 0.02% cholesterol and 6% fat (Lab Diet 5K52; Purina, St. Louis, MO) until two months of age. At two months, mice were randomly assigned to receive a low-cholesterol or high-cholesterol diet. Those that received a high-cholesterol diet were fed D12102N base diet (Research Diets, New Brunswick, NJ) supplemented with 0.15% cholesterol with a constant proportion of fat (4.3%) and other constituents. Mice not assigned to the high-cholesterol diet were continued on the standard low cholesterol laboratory chow diet. Mice were fed their respective diets and had access to water *ad libitum *for periods of 4 or 10 months.

### Measurement of serum cholesterol

At the initiation of dietary manipulations and at the termination of the study, blood samples were taken from the retro-orbital sinus and total plasma cholesterol levels were determined using the Infinity Cholesterol Reagent kit (Thermotrace, Arlington, TX) according to the manufacturer's instructions.

### Tissue processing

Mice were anaesthetized with a mixture of ketamine (100 mg/kg) and xylazine (10 mg/kg) and then sacrificed by transcardial perfusion with cold 1% paraformaldehyde in 0.1 M PBS pH 7.4 (phosphate buffered saline) for 1 min, followed by cold 4% paraformaldehyde in 0.1 M PBS pH 7.4 for 10 min. After perfusion, brains were removed and postfixed in 4% paraformaldehyde for 48 hrs and then transferred to 0.1 M PBS, and stored at 4°C until sectioning. Series of 50 μm-thick coronal sections were cut using a Vibratome.

### Histology and immunohistochemistry

Immunoperoxidase staining was performed on free-floating sections using an antigen retrieval method that utilizes a pepsin digestion treatment which has been previously described [46]. Prior to pepsin treatment, sections were incubated in distilled water for 5 min at 37°C and then transferred to 1 mg/ml pepsin (Dako, Carpinteria, CA) in 0.2 N HCl. Sections were incubated in the pepsin solution at 37°C for 10 min. After washing in PBS for 15 min at 27°C followed by three 10-min washes at room temperature, sections were processed for immunohistochemistry. Sections were pretreated with 10% methanol/1% hydrogen peroxide in PBS for 10 min and then blocked with Tris-buffered saline (TBS; 50 mM Tris-HCl, 0.15 M NaCl, pH 7.6, 0.15 M NaCl)/0.1% Triton X-100/5% goat serum (TBS-TGS) for 1.5 h. After a wash in PBS, sections were incubated with rabbit polyclonal anti-collagen IV antiserum (1:500; Chemicon, Temecula, CA) in TBS-TGS at room temperature overnight. Sections incubated without primary antibody served as controls. Following a wash in PBS, sections were incubated with goat anti-rabbit horseradish peroxidase (HRP)-conjugated secondary antibody (1:500, Santa Cruz Biotechnology, Santa Cruz, CA) for 2 h in TBS-TGS. Staining was visualized using 3,3'-diaminobenzidine in 50 mM Tris-imidazole buffer (pH 7.6). After being mounted on slides, sections were dried overnight and counterstained with 0.5% cresyl violet for 6 min followed by dehydration through a graded series of ethanol solutions (70, 85, 90, 100% for 2 min each). Slides were then treated with Americlear (Fisher, Tustin, CA) for 2 min, followed by xylene for 10 min and coverslipped with Cytoseal 60 (Richard-Allan Scientific, Kalamazoo, MI). Sections were photographed using an Optonics MicroFire true color microscope 1600 × 1200 digital CCD camera (Optronics, Goleta, CA). Digital images were color balanced using Adobe Photoshop 7.0 (Adobe Systems, San Jose, CA).

### Stereologic determination of hippocampal microvascular density, length, and diameter

For analysis of microvascular parameters, every 6th coronal section throughout the hippocampus from LDLR-/- and C57BL6/J mice fed high- and low-cholesterol diets for 10 months was stained with the anti-collagen IV antiserum and counterstained with cresyl violet. The hippocampus was delineated using a stereology workstation, consisting of a modified Olympus BX50 light microscope with a PlanApo objective 2.5× (numerical aperture [N.A.], 0.04) to delineate brain regions and a UPlanApo objective 20× (N.A., 0.8; Olympus, Tokyo, Japan). For counting, a motorized specimen stage for automatic sampling (Ludl Electronics; Hawthorne, NY), CCD color video camera (HV-C20AMP; Hitachi, Tokyo, Japan), and stereology software (StereoInvestigator; MBF Bioscience, Williston, VT) were utilized. Vessel density and length were determined using the "Space Balls" method as previously described [47,48]. Hemispheres with a radius of 30 μm were placed in a systematic-random manner within the sections. The vessel density and length was obtained from the total number of intersections between the hemispheres and vessels (at least 300-400 hits per brain half) as described previously [48]. Vessel diameter was determined by using the "Fast Measure Line" tool of the stereology software. Diameter was taken as the shortest diameter of the outer wall of vessels coming into focus during the Space Ball analysis. Large vessels with a diameter > 30 μm were not considered as representative of the microvasculature and were not included in the analysis for vessel length and density. The number of irregular and pathological microvessels was determined by randomly selecting microvessels using the Space Balls software. Based on visual inspection, an observer blinded to the genotype of the animal, classified each sampled vessel as "normal", "irregular", or abnormally "dilated", "tortuous", or "string-like". Abnormally dilated, tortuous, and string vessels were summed as "pathologic".

### Electron microscopy

Electron microscopy was performed as described previously [49] using the cryofixation/cryosubstitution embedding technique [50]. Three mice from each of the LDLR-/- fed high- or low- cholesterol diets and corresponding genotype controls (C57BL/6J) fed high- or low- cholesterol diets for four months (n = 12 mice total) were anaesthetized and perfused as described above with 4% paraformaldehyde containing 0.125% glutaraldehyde. Tissue was removed and postfixed in the same solution overnight. Brains were then removed and 250 μm-thick coronal sections were cut using a Vibratome and the entorhinal region of the cortex was dissected out. Freeze substitution and low-temperature embedding of the specimens was performed as described elsewhere [50-52]. The slices were cryoprotected by immersion in 4% D-glucose, followed by increasing concentrations of glycerol (from 10% to 30% in phosphate buffer, v/v). Sections were plunged rapidly into liquid propane cooled by liquid nitrogen (-190°C) in a Universal Cryofixation System KF80 (Reichert-Jung, Vienna, Austria). The samples were immersed in 0.5% uranyl acetate dissolved in anhydrous methanol (-90°C, 24 h) in a cryosubstitution AFS unit (Leica, Vienna, Austria). The temperature was raised from -90°C to -45°C in steps of 4°C/h. After washing with anhydrous methanol, the samples were infiltrated with Lowicryl HM20 resin (Electron Microscopy Sciences, Fort Washington, PA) at -45°C. Polymerization with ultraviolet light (360 nm) was performed for 48 hrs at -45°C, followed by 24 h at 0°C. Ultrathin 70 nm sections were cut with a diamond knife on a Reichert-Jung ultramicrotome and mounted on nickel grids using a Coat-Quick adhesive pen (Electron Microscopy Sciences). Grids were examined on a Joel 1200 EX electron microscope (Tokyo, Japan) and imaged with an advantage CCD camera (Advanced Microscopy Techniques, Danvers, MA). Images were adjusted for brightness and contrast using Adobe Photoshop 7.0.

### Determination of apolipoprotein E levels in brain

Mice were sacrificed with carbon dioxide and the brains removed and regionally dissected. Apolipoprotein E (Apo E) levels in brain were determined by quantitative Western blotting with normalization to α-tubulin as previously described [17] using pooled samples of anterior and posterior neocortex.

### Statistical analysis

All data are presented as mean ± the standard error of the mean (S.E.M.). Equality of variance was assessed using the Levene test. Comparisons were made using two-way univariate or repeated measures analysis of variance (ANOVA) as well as unpaired *t *tests. When multiple comparisons were made for all pairs among the four groups, the Tukey HSD procedure was used if the Levene test was not significant (p > 0.05). Otherwise, comparisons for all pairs of groups or selected comparisons were made using unpaired *t *tests with significance determined according to the procedure of Holm [53] to correct for multiple comparisons. If the Levene statistic was significant (p < 0.05) and groups were of unequal sizes, the unpaired *t *tests employed the Welch correction for unequal variances. Statistical tests were performed using the program GraphPad Prism 4.0 (GraphPad Software, San Diego, CA) or SPSS 16.0 (SPSS, Chicago, IL).

## Abbreviations

Aβ: amyloid β peptide; AD: Alzheimer's disease; APP: amyloid precursor protein; ANOVA: analysis of variance; ApoE: apolipoprotein E; CNS: central nervous system; H&E: hematoxylin and eosin; HCD: high-cholesterol diet; ICAM-1: intracellular adhesion molecule 1; LCD: low-cholesterol diet; LDL-C: low-density lipoprotein cholesterol; LDLR: low density lipoprotein receptor; NFT: neurofibrillary tangle; NP: neuritic plaque; PBS: phosphate buffered saline; SEM: standard error of the mean; TBS: Tris-buffered saline; TGS: Triton X-goat serum.

## Competing interests

The authors declare that they have no competing interests.

## Authors' contributions

SF participated in the design of the experiments, carried out the majority of the experimental work, and participated in the data analysis and writing of the manuscript. MAGS, EO, TO, RDG, and DLD participated in different aspects of the collection of the morphological data. WGMJ conducted the EM studies. DFE measured the apolipoprotein E levels. JS participated in the statistical analysis and CS in the analysis of quantitative vascular parameters. SG participated in the interpretation of the results and writing of the manuscript. PRH supervised the collection and analysis of the morphological data and participated in the writing of the manuscript. JDB participated in designing the experiments and drafting the manuscript. GAE participated in experimental design, data analysis, and writing of the manuscript. All authors read and approved the final manuscript.
